# Intra-Articular Injection in Wistar Rats: Standardization and Experimental Validation of a Precise Protocol for Nanomaterial Administration

**DOI:** 10.3390/nano16010009

**Published:** 2025-12-20

**Authors:** Manuel Flores-Sáenz, Belén Chico, Maria C. García Alonso, Celia Clemente de Arriba, Soledad Aguado-Henche

**Affiliations:** 1 University of Alcalá, Department of Surgery, Medical and Social Sciences, Teaching Unit of Human Anatomy and Embryology, Laboratory of Osseointegration and Microscopic Anatomy, Science and Technology Campus, Madrid-Barcelona Highway, Km. 33.600, 28805 Alcalá de Henares, Madrid, Spain; celia.clemente@uah.es (C.C.d.A.); soledad.aguado@uah.es (S.A.-H.); 2 Centro Nacional de Investigaciones Metalúrgicas (CENIM), Consejo Superior de Investigaciones Científicas (CSIC), Avenida Gregorio del Amo, 8, 28040 Madrid, Spain; bchico@cenim.csic.es (B.C.); crisga@cenim.csic.es (M.C.G.A.)

**Keywords:** particle injections, intra-articular rats, Wistar, nanostructures, histology, reproducibility of results, orthopedic procedures

## Abstract

(1) Background: Intra-articular injection is a fundamental technique in preclinical research for evaluating therapeutics and inducing joint disease models in rodents. However, the absence of standardized and validated protocols compromises reproducibility and translational validity. (2) Methods: This study establishes and experimentally validates a refined protocol for precise intra-articular injection in the knee of adult male Wistar rats. The comprehensive procedure specifies anatomical landmarks (medial border of the patellar tendon), instrumentation (27 G needle, 100 µL Hamilton syringe), a maximum volume of 35 µL, and operative verification criteria based on tactile feedback. Experimental validation was performed by administering a suspension of wear particles (2.35 mg/mL) generated from tribocorrosion tests of CoCr surfaces biofunctionalized with graphene oxide-hyaluronic acid (GO-HA) into the left knee of five rats. (3) Results: Histological analysis using the cutting–grinding technique and Toluidine Blue staining confirmed the exclusive intra-articular localization of particles in all injected animals (5/5 success rate). Qualitative assessment revealed abundant particulate distribution within the synovial space, with numerous individual particles and multiple aggregates observed per high-power field, without evidence of extravasation in any case. (4) Conclusions: The protocol demonstrated high intra-operator repeatability and provides a reliable, ethically refined tool for precise intra-articular administration of nanomaterials and for generating robust joint disease models, thereby enhancing reproducibility and animal welfare in preclinical research.

## 1. Introduction

Intra-articular administration constitutes an experimental and therapeutic strategy of considerable interest, as the joint represents an anatomically enclosed environment that enables the accumulation of high local drug, biomaterial, or nanomaterial concentrations while minimizing systemic adverse effects. In this context, the intra-articular route has been extensively employed in animal models both for inducing osteoarthritic processes and for assessing tissue response to the efficacy of various therapeutic formulations^1^. However, the anatomical precision of the approach and procedural standardization critically determine the reproducibility of outcomes and the validity of the resulting models. The possibility of delivering nanomaterials or biomolecules in a controlled fashion directly into the articular space confers particular translational relevance to this route in studying regenerative therapies and synovial environment modulators.

Despite the widespread use of intra-articular injection in experimental rodent models, the scientific literature exhibits marked heterogeneity in the protocols described. Technical factors—including the anatomical access point, insertion angle, needle type, administered volume, and confirmation of correct intra-articular placement—vary considerably across studies, potentially leading to substantial differences in injected material distribution, tissue response extent, or even the occurrence of false-negative results [1]. Consequently, anatomical precision and operative standardization of the approach are essential prerequisites for ensuring comparability of results across investigations. While several studies have utilized the intra-articular route in rat models of inflammation or joint pain, such as collagenase [1,2] or complete Freund’s adjuvant [3] injection, detailed anatomical descriptions and technical validation of these procedures remain scarce or altogether absent.

This methodological gap is particularly evident in the rat, one of the most widely employed species in biomedical research owing to its intermediate size, anatomical accessibility, and suitability for biocompatibility and pharmacokinetic studies. Even authoritative national references such as the revised edition of *Ciencia y Tecnología del Animal de Laboratorio* [4], regarded as the most comprehensive Spanish-language manual on laboratory animal science, lack a detailed technical description of the intra-articular approach in this model. This absence underscores the need for establishing standardized anatomical protocols enabling precise and reproducible application of this technique. This methodological deficiency has also been acknowledged in international guidelines on refinement and reproducibility [5,6], which stress the importance of accurately describing the anatomical and technical parameters of intra-articular administration in small animal models. In accordance with ARRIVE 2.0 guideline recommendations [6], comprehensive and transparent description of experimental procedures represents an essential requirement for ensuring scientific reproducibility and animal welfare in preclinical research.

This methodological deficiency is particularly critical in the field of nanomaterial-based joint therapies and diagnostics. Intra-articular administration of nanoparticles, nano-structured drug delivery systems, or particulate biomaterials demands millimeter-level precision due to several factors: (a) the typically low therapeutic doses (µg to mg range), (b) the need for localized distribution to assess specific biointeractions at the nano-bio interface, and (c) the direct impact on the reproducibility of biocompatibility and biodistribution studies [5,6]. A non-validated injection protocol introduces unacceptable technical variability, jeopardizing the translational potential of novel nano-therapeutics and the reliability of preclinical models for nanomaterial safety assessment.

In previous work by our group [7], intra-articular injection was employed to evaluate hematological and tissue responses following administration of nanomaterials derived from CoCr alloy surfaces biofunctionalized with graphene oxide and hyaluronic acid (GO–HA). Although the technique proved feasible and safe, the methodological description was necessarily succinct and did not address in detail the technical and anatomical aspects of the procedure. Building on this prior experience [7] and with the aim of providing a comprehensive technical guide, the present work seeks to exhaustively describe the intra-articular injection technique in adult male Wistar rats, including experimental conditions, anatomical landmarks, instrumentation, and safety considerations required to ensure proper execution. This work further intends to establish a robust methodological foundation serving as a reference for future investigations aimed at evaluating its validity, reproducibility, and applicability across different experimental models.

## 2. Materials and Methods

### 2.1. Intra-Articular Injection Procedure in Wistar Rat Knee

#### 2.1.1. Ethical Justification and 3R Principles 

The methodological design adheres to the international principles of Replacement, Reduction, and Refinement (3Rs) [8] and the European Union Directive 2024/1262 [9], and in Spain by Royal Decree 53/2013 [10]. The use of the minimum number of animals ensuring sufficient statistical power is recommended, avoiding unnecessary repetitions and prioritizing the acquisition of valid information per animal. Refinement is applied through anesthesia and analgesia protocols, humane endpoint criteria, and environmental enrichment. Compliance with ARRIVE 2.0 guidelines [6] is considered mandatory to ensure reproducibility and transparency in future experimental applications.

#### 2.1.2. Animals and Husbandry Conditions

The use of adult male Wistar SPF (Specific Pathogen Free) rats weighing 250–300 g and aged 70 ± 1 days is recommended. Specification of a single strain, sex, and age/weight range is essential in technical standardization protocols, as it minimizes biological confounding factors and ensures procedural reproducibility.

Animals should be housed in accredited facilities under the following husbandry conditions:

- Temperature: 20–26 °C (with variations ≤ 2 °C/day)
- Relative humidity: 40–60%
- Photoperiod: 12 h light/12 h dark
- Ventilation: 10–15 air changes/hour
- Cages: transparent polycarbonate, ≈1500 cm^2^ for ≤5 rats
- Bedding: autoclaved wood shavings, changed 2–3 times/week
- Enrichment: tubes, nesting material, and toys
- Food and water: ad libitum; irradiated pellets and autoclaved filtered water
- Handling: by personnel trained in non-aversive techniques^4^

#### 2.1.3. Asepsis and Material Preparation

All solutions to be inoculated must be prepared under aseptic conditions through sterile microfiltration using a 0.22 µm PVDF filter (MilliporeSigma, Burlington, MA, USA) and handling in a laminar flow cabinet. Suspensions are warmed to body temperature (≈37 °C) prior to administration to prevent hypothermia or thermal shock [4]. Each needle and syringe is changed after each animal to prevent cross-contamination.

Technical note: 0.22 µm filters constitute the international standard for filtration sterilization in aqueous or colloidal solutions, eliminating bacteria and fungi without affecting the stability of nanomaterials or biocompatible molecules [11].

#### 2.1.4. Intra-Articular Injection

The protocol is designed for administering sterile solutions or suspensions of low viscosity (<10 cP) into the synovial cavity of the knee, ensuring homogeneous material distribution without capsular distension.

#### 2.1.5. Anesthesia and Monitoring

Rats are anesthetized with 5% isoflurane for induction (O_2_ flow 1 L/min) (Figure S1A) and maintained at 2% throughout the procedure (Figure S1B). Anesthetic depth is verified by the absence of pedal and corneal reflexes. Body temperature is maintained at 37 °C using a heating pad with continuous monitoring. Inhalational anesthesia administration is the preferred method due to its rapid reversal and low physiological impact [4,5,6,7,8,9,10].

#### 2.1.6. Injection Material

A Hamilton 710LT SYR syringe (100 µL) (Hamilton Company, Reno, NV, USA) (Figure S2) coupled to a 27 G × ½″ (0.4 × 12 mm) stainless steel needle (Nipro Medical Corporation, Osaka, Japan) is used, in accordance with recommendations by Wolfensohn and Lloyd^11^ and the Spanish Society for Laboratory Animal Science (SECAL)^4^ (Figure S3).

The Hamilton 710LT syringe was selected for its high volumetric precision (±1% accuracy) in the microliter range, which is critical for the reproducible administration of nanomaterials. While insulin syringes (27 G or 30 G) are commonly used for rodent injections, their typical volumetric variability (±5–10% in the 30–50 µL range) could introduce significant dosing errors when administering low-dose nanomaterials or when exact volume reproducibility is required for comparative studies. The use of this precision syringe minimizes a potential source of technical variability in nanomaterial biodistribution and response studies. For centers without access to sterilized Hamilton syringes, pre-sterilized disposable versions are commercially available.

#### 2.1.7. Anatomical Positioning and Surgical Technique

**1.**  **Disinfection:** The anterior knee area was shaved and cleaned with 70–96% ethanol (Figure S4A,B)
**2.**  **Anatomical Positioning**: The animal was placed in dorsal decubitus, with the limb in 90-degree flexion (Figure S5).
**3.**  **Landmark Identification**: The anterior tibial tuberosity and patellar tendon were identified by palpation. The interarticular line was similarly identified using the operator’s fingernail (Figure S6).
**4.**  **Surgical Approach:** The approach was performed in the medial femorotibial region (stifle), with penetration perpendicular to the sagittal plane and slightly oblique to the transverse plane, allowing direct access to the common synovial space of the femoropatellar and femorotibial cavities without damaging ligamentous or neurovascular structures [1].
**5.**  **Entry Point:** Medial border of the patellar tendon, above the tibial plateau.
**6.**  **Insertion:** The needle was introduced perpendicular to the skin, with a slight cranial inclination, until a sudden loss of resistance was perceived. The needle is inserted perpendicular to the skin (90° to the sagittal plane) with a slight cranial inclination (10–15°), advancing 3–4 mm until a sudden loss of resistance is perceived upon traversing the joint capsule. This depth is appropriate for rats weighing 250–300 g.

**Figure 1:**
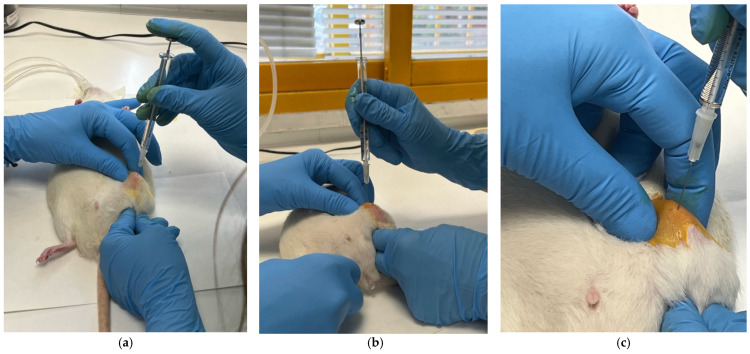
Sequence of the intra-articular approach. (**a**) Medial entry point to the patellar tendon. (**b**) Needle insertion directed towards the synovial space. (**c**) Needle insertion was performed at 90° to the sagittal plane with 10–15° cranial inclination, advancing 3–4 mm to reach the synovial space in rats weighing 250–300 g.

The procedures described in points 4, 5, and 6 are detailed in Figure 1:

**7.**  **Injection:** A maximum volume of 35 µL of sterile suspension should be administered at a slow rate (≈10 s) to ensure homogeneous distribution and avoid synovial overpressure. The physiological capacity of the synovial space in rats is estimated to be 25–35 µL [11].
**8.**  **Verification of Intra-Articular Location:**
  - Sudden loss of resistance upon traversing the capsule
  - Absence of bloody reflux upon aspiration
  - Absence of subcutaneous extravasation during injection
  - Smooth injection flow without lumps or anomalous resistance
**9.**  **Withdrawal and Subsequent Control:** The needle should be withdrawn slowly while applying gentle pressure with sterile gauze to prevent leakage.
**10.**  **Immediate Check:** The absence of extravasation and preserved joint mobility should be verified.

#### 2.1.8. Losses, Bleeding, and Post-Injection Control

Animals are maintained on a heated surface until full recovery.

Preventive postoperative analgesia (meloxicam 1 mg/kg s.c. every 24 h for 3 days) and daily welfare monitoring are recommended, in accordance with RD 53/2013 [10] and the Spanish Society for Laboratory Animal Science (SECAL) recommendations [4] (2018).

#### 2.1.9. Postoperative Control

Light pressure is applied to the puncture point to prevent leakage or bleeding, and hemarthrosis or visible extravasation is ruled out.

Observation for 24 h post-injection allows for the identification of non-specific inflammatory reactions or signs of pain, adjusting analgesia if necessary [12].

#### 2.1.10. Euthanasia and Traceability

Euthanasia must be performed using sodium thiopental (100 mg/kg i.v., anesthetic grade) or an approved equivalent, following the recommendations of Clarkson et al. [13] and RD 53/2013 [10]. For the animals in this validation study, euthanasia was performed via CO_2_ inhalation.

The procedure must be documented with a record of the batch, route, and time of administration.

Biological waste is managed in accordance with European biosafety regulations.

#### 2.1.11. Recording and Traceability Recommendations (ARRIVE Checklist)

It is recommended to attach a simplified ARRIVE checklist [6] to the procedure, ensuring (see Table 1):

- Animal identification (species, strain, sex, age, weight, origin).
- Housing conditions and number of animals per group.
- Complete details of anesthesia, instrumentation, and injected volume.
- Intra-articular verification procedures and postoperative observation.
- Ethical compliance and references to institutional licenses.

The authors or institutions reproducing this procedure will be responsible for its ethical and legal adaptation to the applicable regulatory framework in their country.

#### 2.1.12. Additional Considerations

While the present protocol was developed using low-viscosity aqueous formulations, such as the dispersion of wear particles from CoCr surfaces with graphene oxide-hyaluronic acid (GO–HA) in our previous work [7], it is crucial to consider the impact of this physicochemical property on the execution of the technique. The scientific literature demonstrates that the administration of more viscous formulations (e.g., hydrogels, biomaterial concentrates) presents additional technical challenges [5,14]. High viscosity can significantly increase the injection force, requiring larger gauge needles that may increase tissue trauma [15]. Furthermore, it can alter the distribution kinetics within the synovial space, favoring the formation of local deposits rather than a homogeneous distribution, which directly influences the biological response and the interpretation of the results [16]. Therefore, it is strongly recommended to characterize and report the viscosity of any new formulation that deviates from a standard aqueous fluid. For viscous formulations, pilot studies characterizing the injection force and verifying the intra-articular distribution are essential.

### 2.2. Experimental Validation of the Protocol

To validate the anatomical precision and operational reproducibility of the protocol, a verification study was conducted. A suspension of wear particles obtained from tribocorrosion tests of cobalt–chromium (CoCr) alloy surfaces, previously functionalized with graphene oxide–hyaluronic acid (GO-HA), was selected as a model load. Briefly, the tests were performed on a pin-on-disk tribometer (Microtest, Madrid, Spain) with an applied load of 5 N, at 120 rpm, and over sliding distances of 30,000 m, in a cell containing an aqueous solution of hyaluronic acid (3 g/L, pharmaceutical grade). The generated wear particles, after collection, isolation, and washing in distilled water, were used at a concentration of 2.35 mg/mL in physiological saline (0.9% NaCl). This formulation, whose generation and characterization are described in our previous work [7], constitutes a model for the intra-articular injection of sub- and microparticles into the joint cavity. Its choice as a tracer is based on its biological relevance for biocompatibility and safety studies of biomaterials, and its suitability as a visual marker for the histological confirmation of the intra-articular location, given its distinctive particle size distribution, morphology, and composition.

#### 2.2.1. Animals and Ethical Considerations

The validation study was performed on five adult male Wistar rats (250–300 g). This sample size is considered adequate and sufficient for the technical validation of in vivo procedures, consistent with the Reduction principle of the 3Rs by employing the minimum number of animals necessary to obtain a scientifically valid result on the administration’s precision. This criterion aligns with the established methodological literature for the validation of experimental techniques, where sample sizes in this range allow for the demonstration of a procedure’s feasibility and precision [17]. All experimental procedures, including this validation study, were reviewed and approved by the Regional Clinical Research Ethics Committee of Madrid (CEIC-R) with reference number PROEX 232/19.

#### 2.2.2. Administration

A single experienced operator performed the intra-articular injection in the left knee of the animals, strictly following all steps of the standardized protocol described herein. The decision to employ a single operator for this initial technical validation phase is methodologically sound, as it allows for the assessment of the method’s intra-operator reproducibility per se, eliminating inter-operator variability as a confounding factor at this technique-establishment stage [17]. The evaluation of inter-operator reproducibility among several surgeons constitutes a subsequent external validation stage, equally crucial for the multicentric implementation of the protocol.

#### 2.2.3. Histological Processing

**Figure 2:**
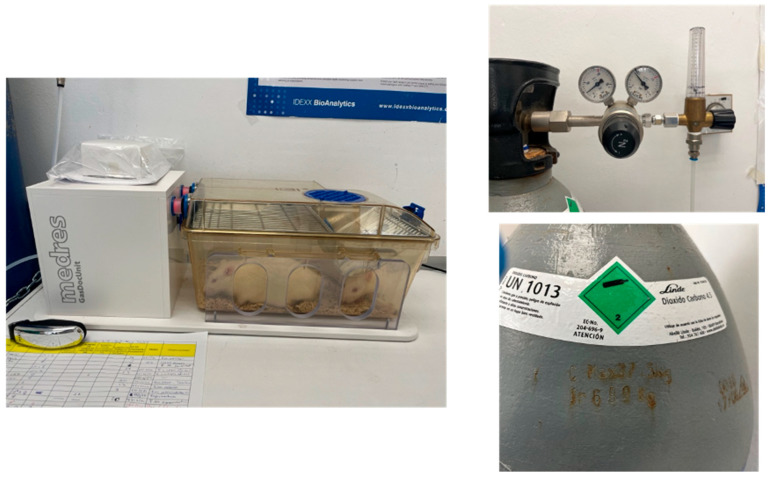
Equipment and procedure for euthanasia via CO_2_ chamber and subsequent blood extraction.

**Figure 3:**
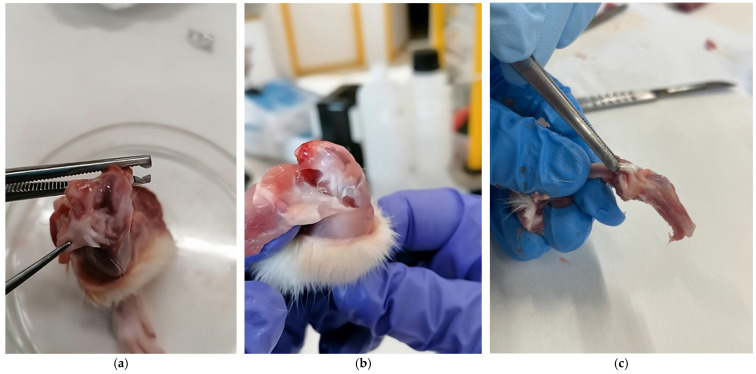
(**a**–**c**) Initial processing of the post-euthanasia joint. Dissection and knee sample obtention.

After a postoperative period of 24 h, the animals were euthanized via CO_2_ inhalation in a standard laboratory euthanasia chambertraction (Figure 2), the left knee was obtained from each rat for fixation and subsequent histological study (Figure 3). It was decided to extract one knee from each specimen to prevent greater suffering to the animal.

For sample obtention, a protocol based on the method of Donath and Breuner [18] was applied, with the modifications established by the Osteointegration Laboratory of the Department of Surgery, Medical and Social Sciences at the Faculty of Medicine and Health Sciences of the University of Alcalá [19].

The method developed by Donath and Breuner [18] is based on the cut-and-grinding technique. Its objective is to obtain fine final sections (approximately 50 µm thick) from fragments that cannot be cut or processed conventionally (such as through paraffin embedding). Therefore, the protocol used in this research is proposed as an alternative to traditional bone tissue processing for the following reasons:

Most hard tissues cannot be processed by conventional techniques (such as cryo-paraffin and hard-section cutting) due to the prior decalcification they must undergo.

The possibility of processing bone without the need to decalcify the sample enables:

- Successful manipulation of the sample for its preservation.
- Subsequent histological study of the sample (with or without an implant).
- Study of the bone-implant interface.

This protocol consists of the following phases:

**1.**  **Fixation:** Once the knees were obtained, sample fixation was performed. The objective of the fixation process is to halt autolysis and tissue degradation processes. For this purpose, a 4% formalin solution (histological grade), buffered to pH 7 with sodium phosphate, was used to avoid premature decalcification of the samples. The use of 4% formalin was chosen due to its optimal penetration into hard and dense tissue. For adequate sample fixation, a volume of fixative at least 10–20 times the sample volume is required. For samples of this thickness, it was necessary to extend the fixation time to at least 48–72 h.
**2.**  **Dehydration and Embedding in Glycol Methacrylate (GMA):** Subsequently, sample dehydration was carried out. The dehydration of bone samples fixed in 4% formaldehyde solution (histological grade) requires increasing concentrations of glycol methacrylate (GMA, Sigma-Aldrich, Darmstadt, Germany). Since formaldehyde is water-soluble, whereas GMA is hydrophobic, using 100% GMA directly would impede the gradual removal of formaldehyde, which could generate trapped bubbles in the sample and affect the quality of the embedding. For the final step of increasing GMA concentrations (100% GMA), the samples were maintained for 48 h to ensure successful embedding. The primary reason for performing an intermediate embedding in GMA instead of directly in Technovit 7200 (Kulzer GmbH, Hanau, Germany) was cost minimization, as well as the fact that GMA tends to acquire a yellowish color, which would hinder subsequent sample study.
**3.**  **Embedding in Technovit^®^ 7200:** The sample was embedded in Technovit^®^ 7200, starting with a GMA/Technovit^®^ 7200 ratio of 50:50. Embedding in this medium provided adequate infiltration of the cartilage. After approximately 48 h, the Technovit^®^ 7200 concentration was increased to 100%, with the aim of remaining embedded for at least fourteen days. In this case, the samples remained embedded for 90 days. As a step prior to photopolymerization, the samples were placed in embedding molds to secure their final position.
**4.**  Photopolymerization: Subsequently, photopolymerization of the already embedded samples was carried out. This procedure aims to achieve the solidification and hardening of the embedding medium. This protocol includes a two-phase photopolymerization process:
  - Stage 1: White light is used, and the temperature is maintained around 40 °C. In this way, the embedding medium becomes intensely polymerized.
  - Stage 2: Blue light is used at a higher intensity. The embedding medium that has infiltrated the tissue becomes completely polymerized.

Although using a two-phase polymerization procedure is slower, it prevents bubble formation. After completing the two previous stages, the samples were placed in an incubator at 50 °C for 13 h to complement the photopolymerization. Supplemental polymerization with benzoyl peroxide was not required.

**5.**  Cut-and-Grinding (EXAKT^®^ System): For this purpose, the EXAKT^®^ system (Vertriebs, Norderstedt, Germany) was used. The EXAKT^®^ cut-and-grinding system consists of three main components: the cutting unit, the parallel precision guide, and the water-jet cooling system. The cutting unit is based on the principle of “serrated fillets” and its speed can be adjusted according to the material’s hardness. The cutting saw blade has a cutting edge coated with diamond particles. The procedure was as follows:
  - Block Preparation and Obtaining a Parallel Surface: Once the polymerized tissue block was extracted, preliminary grinding was performed. To achieve a smooth surface, the opposite side of the block was mounted on a guide with Technovit^®^ 4000.
  - Preparation of the Surface of Interest: The block mounted on the guide was placed in the vacuum apparatus attached to a parallel micro-grinding system. The surface was successively polished with 1200-grit and subsequently 4000-grit sandpaper until the smoothest possible surface was obtained.
  - Obtaining Sections: Several cuts of 400 µm thickness were made using a cutting unit with a diamond-coated saw band. The cuts were made perpendicular to the longitudinal axis of the tibial diaphysis along a transverse plane. Each obtained section was ground with the EXAKT^®^ System grinder until a final thickness of approximately 50–100 µm was achieved. The final sample thickness was measured by calculating the difference between the thickness of the slide with the sample and the thickness of the slide alone.

#### 2.2.4. Histological Staining

For a complete histological characterization of the joint, Hematoxylin-Eosin (H&E) and Toluidine Blue (TB) staining were used concurrently. This practice, established in osteoarticular histology [19], optimizes the morphological and structural information obtained from the samples by assigning a specific analytical objective to each stain:

Hematoxylin-Eosin (H&E) staining was specifically used for reference anatomical description and the assessment of general tissue architecture. As noted by Flores-Sáenz [19], H&E is the most common stain in laboratories and allows for clear observation of bone marrow cells or inflammatory cells, thereby providing the essential cytoarchitectural context for all articular structures (synovial membrane, capsule, bone, etc.).

Toluidine Blue (TB) staining was reserved for the specific analysis of particle localization and the evaluation of articular cartilage. This choice is based on its superiority in staining cartilaginous components, as ‘proteoglycans stain a more intense blue due to the metachromatic reaction with acid mucopolysaccharides’ [19], creating the optimal contrast necessary to identify exogenous particles within the joint space.

This dual strategy ensures that the anatomical description is comparable with the extensive existing literature (mostly based on H&E), while the analysis of specific findings benefits from the specialized sensitivity of TB.

## 3. Results

### 3.1. Characterization of Wear Particles and Pre-Injection Analysis

To unequivocally identify the particles within the joint, their morphology was first characterized in the pre-injection state. For this purpose, an extension of the particle suspension was prepared on a glass slide, which was covered with a coverslip to avoid contamination.

**Figure 4:**
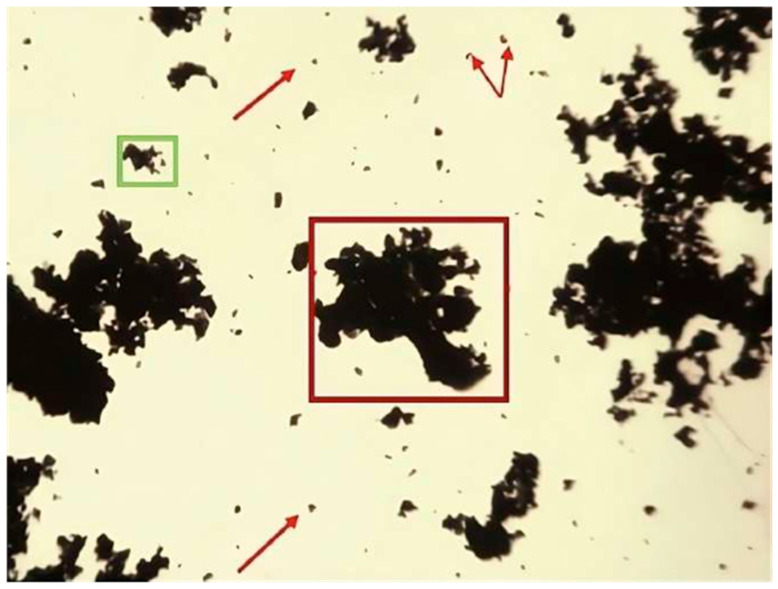
Characterization of wear particles and their arrangement (50× magnification using a binocular loupe). Squares indic standard laboratory binocular loupeaticles forming deposits of different sizes (green, red). Arrows point to isolated graphene particles.

As shown in Figure 4, isolated wear particles with their characteristic polygonal shape were observed. Likewise, particle agglomerations were identified, with a morphological appearance similar to that documented in our previous work [7]. According to the results of the semi-quantitative analysis performed by ICP-OES of the particle dispersion, described in [7], the elements found in the highest concentration and in virtually the same proportion were Co and Cr, both originating, especially, from the base material. The Si element originates from the APTES compound, used as an intermediary for the covalent bonding of the GO to the CoCr surface. The size distribution of the wear particles according to the Dv10, Dv50, and Dv90 percentiles are 7.15 μm, 25 μm, and 78.2 μm, respectively. This means that the volume of filtered material in the form of nanoparticles is marginal, while a considerable proportion has been collected as larger particles. Due to their larger size, the microparticles are recognized more rapidly by cells of the immune system, which provokes a more localized response and favors their retention in the intra-articular cavity [20]. Therefore, this particle size is ideal, as their permanence in the cavity allows for more precise verification of the correct execution of the surgical technique.

### 3.2. Anatomical Reference Description of the Wistar Rat Knee

**Figure 5:**
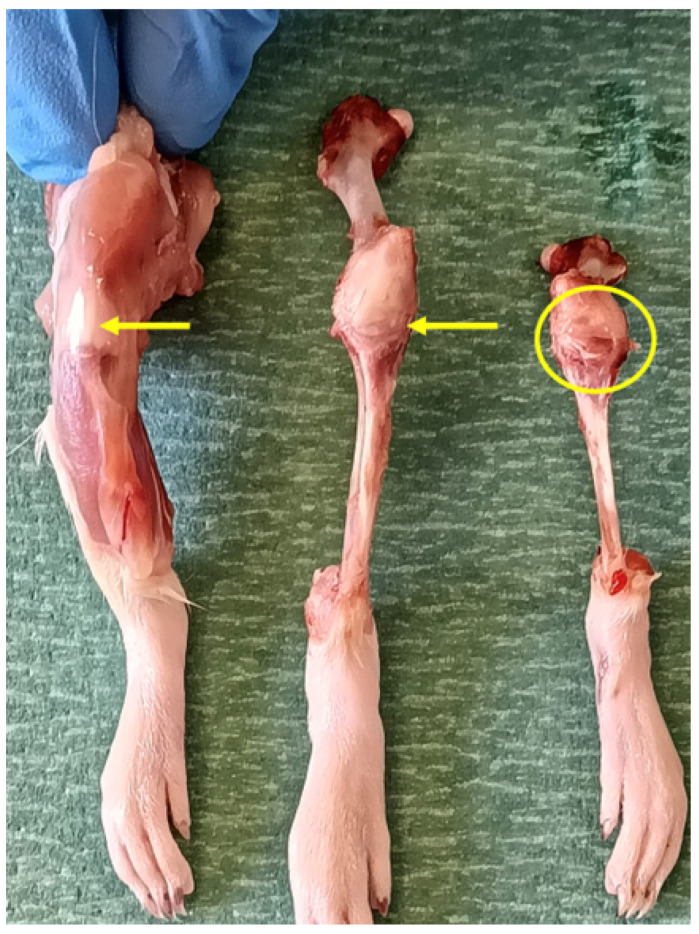
Sequential macroscopic dissection of the Wistar rat knee illustrating the anatomical approach for intra-articular injection. The sequence (left to right) shows: the medial view of the intact knee with the patellar ligament indicated (yellow arrow); the isolation of the patellar ligament (yellow arrow) following dissection; and the transverse section of the ligament, revealing the intra-articular synovial space (yellow arrow) targeted during injection.

**Figure 6:**
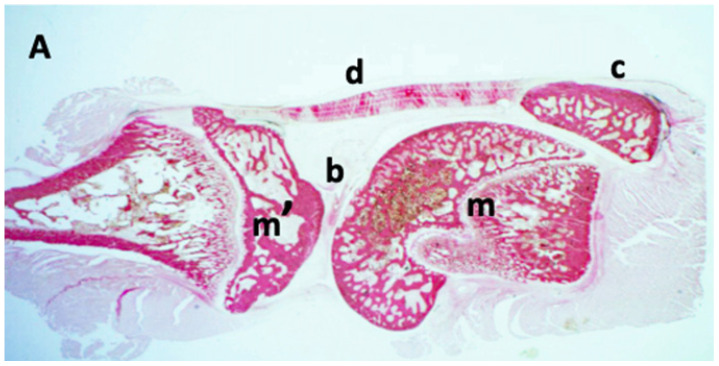
Parasagittal section of the knee (I). Staining: Hematoxylin-eosin. 8× magnification in binocular loupe. Description of a standard laboratory binocular loupe l anatomy: (A) The tibia is observed on the left side. On the right side, the femoral condyle is visible. Both structures are separated by the interarticular line (structure [b]). In the anterior part of the joint in the image, the patellar tendon (structure [d]) attached to the patella (structure [c]) can be seen inserting into the anterior part of the proximal extremity of the tibia. Note the presence of bone growth metaphyses in both the femur (structure [m]) and the tibia (structure [m’]).

Prior to the analysis of the results, the macroscopic anatomy of the Wistar rat knee is described to facilitate the understanding of the findings (Figure 5). A healthy joint exhibits a defined architecture where the tibia and femur articulate, with the joint space delineated by the synovial capsule and periarticular soft tissues. In the macroscopic images obtained with a standard laboratory binocular loupe, tissue structures are distinguished by their morphology, topography, and natural tissue contrast, enabling the identification of the main articular components (Figure 6).

### 3.3. Intra-Articular Localization of Wear Particles

To provide quantitative support for the reproducibility of the injection technique, we performed particle distribution analysis in standardized high-power fields (HPF, 40×) from Toluidine Blue-stained sections. The success rate of intra-articular injection was 5/5, with an exact 95% confidence interval of 47.8% to 100% (Clopper-Pearson method). The distribution of particles was consistent across animals, with a mean of 8.4 ± 1.1 HPFs containing particles per section (mean ± SD, *n* = 5). Table 2 summarizes the individual animal outcomes.

**Figure 7:**
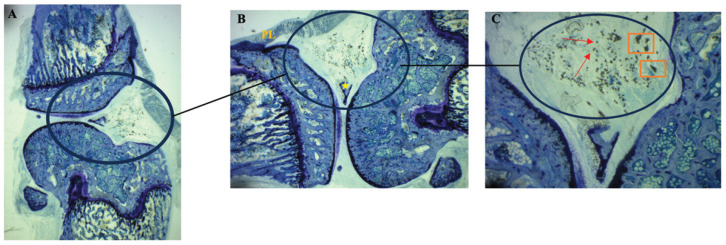
Parasagittal section of a rat knee stained with Toluidine Blue showing particle distribution in the joint space 30 days post-injection. (**A**) Overall view at 12.5× of the knee joint architecture; (**B**) Detail at 16.5× of the joint space (circle) showing the articular meniscus (yellow star). The patellar ligament (PL) serves to orient the anterior anatomical position towards the top of the image. Note that Toluidine Blue staining colors young bone tissue dark blue; (**C**) Higher magnification detail (40×) of the region indicated in (**B**), showing individual particles (red arrows) and nanoparticle aggregates (orange boxes) within the joint tissue.

Histological analysis of the knee sections stained with Toluidine Blue confirmed the presence of exogenous material in the joint cavity of all injected animals (n = 5). In Figure 7 (at different magnifications), particle aggregates with morphology and size consistent with the previously characterized wear particles can be observed. Qualitative analysis of the sections revealed an abundant distribution of particulate material in the synovial space. In representative fields at 40× (Figure 7C), numerous individual particles (more than 10 per field) along with multiple aggregates of variable size were identified. This distribution pattern was consistently observed in all five analyzed animals (intra-articular administration success rate: 100%). These aggregates were located exclusively within the synovial space, in close proximity to the synovial membrane and articular cartilage. No evidence of particle extravasation into the subsynovial tissue, periarticular space, or bone parenchyma was observed.

## 4. Discussion

### 4.1. Experimental Evidence of Precision and Refinement

The selection of the access point, instrumentation, and injection volumes presented in Section 1 is based on anatomical optimization and the principle of refinement. However, it is the experimental validation conducted (Section 2) that provides the crucial evidence of its efficacy. The histological confirmation that the CoCr-GO-HA wear particles were located exclusively within the synovial space in all 5 injected animals tangibly corroborates that the proposed approach—through the medial border of the patellar tendon using a 27 G needle—is anatomically precise.

It should be noted that, alongside isolated particles, larger deposits or conglomerates were observed. While their presence confirms the intra-articular location, the formation of these aggregates can be plausibly attributed to an artifact of the histological processing. The dehydration and inclusion phases in synthetic resins (GMA and Technovit^®^ 7200) constitute an aggressive physico-chemical process that can alter the stability of the injected suspension, promoting particle agglomeration. Therefore, while the intra-articular localization is the primary finding validating the technique, the exact physical distribution (degree of agglomeration) may not faithfully reflect the state immediately post-injection, without diminishing the main conclusion regarding the precision of the administration.

This finding specifically validates our operative criteria for verifying intra-articular localization. We prioritized the “sudden loss of resistance” and the absence of reflux over the systematic aspiration of synovial fluid, a risky practice in rodents. The homogeneous intra-articular distribution of the particles, confirmed through rigorous cutting–grinding processing, demonstrates that this combination of tactile and visual criteria is a reliable and sufficient indicator, minimizing iatrogenic trauma without compromising the validity of the procedure.

Furthermore, the use of a volume of 35 µL, corresponding to a total particle mass of 70.5 µg, theoretically justified by the physiological capacity of the joint, was demonstrated to be operationally adequate [21]. No extravasation was observed, indicating that this volume can be safely administered without generating the capsular overpressure and pain associated with larger volumes [21,22], thus constituting a technically and ethically robust choice. Our selection of 35 µL as the maximum injectable volume is justified by physiological capacity and supported by empirical leakage studies. This volume aligns with the safe range identified in systematic optimization studies. Aytekin et al. [22] demonstrated that intra-articular volumes >50 µL in rat knees caused periarticular leakage in approximately 40% of cases, whereas volumes ≤35 µL minimized this risk. Corroborating this, the magnetic resonance imaging (MRI)-based study of Wang et al. [21] documented leakage with volumes ≥30 µL, but not with 20 µL, leading to a recommended range of 20–30 µL. Our volume of 35 µL therefore represents a conservative upper bound within this evidenced-based safe range, allowing administration of sufficient material for detection while minimizing leakage risk. Minimizing extravasation is particularly critical when administering nanomaterials or bioactive agents, as leakage into periarticular tissues can provoke confounding inflammation, alter local biodistribution, and compromise the interpretation of biocompatibility or therapeutic efficacy studies.

In this study, the absence of extravasation was confirmed by macroscopic observation immediately after injection and, definitively, by histological examination at the tissue level. While non-invasive imaging techniques such as X-ray or micro-CT could provide complementary, longitudinal data on the distribution of an injected solution in future studies, the histological methods employed here (cutting–grinding combined with Toluidine Blue staining) constitute a direct and high-resolution gold standard for verifying the precise intra-articular localization of particulate material and ruling out periarticular leakage.

### 4.2. From Description to Validation: Implications for Nanomaterials and Advanced Therapies

Although intra-articular injection was used in our previous work [7], the lack of explicit validation of the technique constituted a methodological limitation. This work overcomes that deficiency. The successful and confirmed administration of wear particles—a complex material with potential application in joint implantology—elevates this protocol from a mere description to a validated methodological tool, whose rigor is a prerequisite for any reliable translational research in the field of intra-articular therapies.

The ability to reproducibly and verifiably administer nano/micro-material loads into the synovial cavity is a non-negotiable prerequisite for studies of biocompatibility, biodistribution, and efficacy. Any variability in administration introduces an insurmountable technical bias. Our validation lays the methodological groundwork for future research to confidently attribute the observed biological effects to the interaction of the material of interest with the joint environment, and not to artifacts of an incorrect or inconsistent administration.

### 4.3. Anatomical Considerations for Translational Research

A relevant consideration for the translational value of this rodent protocol is the anatomical comparability between the rat and human knee. The fundamental tri-compartmental anatomy (femoropatellar, medial and lateral femorotibial) is conserved. Key landmarks such as the patella, patellar tendon, and tibial plateau are present and palpable in both species, making the principle of a medial parapatellar approach directly relevant. However, critical differences exist and must inform protocol design. The rat knee is significantly smaller, with a synovial capacity of ~25–35 µL compared to the human adult capacity of 100–200 mL This dictates the strict micro-volume administration we describe. Furthermore, the angle of needle insertion and the depth required to penetrate the capsule are proportionally much smaller. Our protocol accounts for these scale differences by specifying precise metrics (e.g., 3–4 mm depth, 10–15° angle) for the 250–300 g rat. Therefore, while the anatomical principles are transferable, the technical parameters are species-specific and cannot be directly extrapolated without validation.

### 4.4. Systematic Comparison with Existing Intra-Articular Injection Protocols

To objectively assess the methodological heterogeneity in the field and substantiate the need for standardization, we performed a systematic comparison of key technical parameters across representative intra-articular injection protocols for the rodent knee (Table 3). This analysis encompasses variations in anatomical landmarks, needle specifications, injection volumes, and methods for verifying intra-articular placement. The comparison reveals a notable lack of consensus, particularly regarding the precision of anatomical descriptions and the justification of injected volumes—factors that directly influence the reproducibility, validity, and translational potential of preclinical joint studies.

Regarding the application of this protocol for constructing robust joint disease models, we acknowledge that the present validation used chemically inert wear particles as tracers. This approach constitutes a **necessary and methodologically rigorous first step**. Before introducing bioactive, disease-inducing agents (e.g., MIA or collagenase), it is imperative to first **establish and verify the precision and reproducibility of the delivery technique itself**. Variability in the placement of such agents is a recognized source of inconsistency in preclinical OA models, leading to unpredictable lesion severity, location, and consequently, high pathological heterogeneity [1,23,24,25]. Our protocol directly addresses this issue by providing a standardized method with **histologically confirmed 100% success in intra-synovial delivery**. When applied to administer agents like MIA, this precision ensures the injurious stimulus originates reliably within the joint space, minimizing periarticular extravasation that can cause confounding inflammation or necrosis. This should result in a more consistent onset and progression of pathology, reducing inter-animal variability and improving the reproducibility and translational validity of the model. Future studies will explicitly employ this validated protocol to induce OA, enabling a direct comparative analysis of pathological outcomes and model consistency against those generated by conventional, less standardized injection techniques.

Our systematic comparison underscores the need for precision in intra-articular delivery. This requirement gains further significance considering contemporary OA research, which is shifting focus from studying tissues in isolation to understanding the complex molecular crosstalk between joint compartments. For example, recent single-cell evidence positions the synovium as an active driver of OA progression through specific signaling pathways with the meniscus [26]. In this context, a standardized and precise injection protocol becomes fundamental, as it ensures that experimental agents (whether therapeutic nanomaterials or disease-inducing molecules) are delivered accurately to the synovial space, enabling the faithful study of this synovium-driven communication and its modulation. Conversely, inaccurate injections that cause periarticular leakage could generate confounding inflammation, obscuring the very inter-tissue signaling networks under investigation. Thus, the methodological rigor validated here provides the technical foundation required for the next generation of studies aimed at dissecting and targeting the multicellular pathophysiology of OA.

### 4.5. Limitations and Future Perspectives

It is important to recognize the limitations of this study. Firstly, the validation was performed in adult male Wistar rats. Translation to other strains, sexes, ages, or smaller species will require specific adjustments, particularly in the injection volume, which must be validated in the same manner.

Secondly, the verification of injection success relied on terminal histology. Although this provides definitive tissue-level evidence, the incorporation of non-invasive imaging modalities (e.g., contrast-enhanced X-ray or micro-CT) in future work would allow for longitudinal tracking of material distribution without the need for sacrifice.

Finally, the validation of inter-operator reproducibility is a crucial subsequent step. Although the use of a single expert operator was the correct strategy to establish the inherent precision of the protocol (repeatability), future studies must quantify its success when performed by multiple operators with different levels of experience, which would confirm its robustness and ease of adoption.

Despite these limitations, this work establishes a solid and, most importantly, empirically supported methodological basis for improving the precision, reproducibility, and animal welfare in preclinical research involving intra-articular administration.

## 5. Conclusions

This work establishes a standardized and experimentally validated protocol for intra-articular injection in the knee of the Wistar rat, addressing a significant methodological gap in the preclinical literature. Based on the objective results obtained, the following conclusions can be drawn.

A detailed protocol for intra-articular injection in the knee of the Wistar rat has been developed and described. The procedure specifies anatomical landmarks (medial border of the patellar tendon), instrumentation (27 G needle, 100 µL Hamilton syringe), a maximum volume of 35 µL, and operative criteria for verification.

Experimental validation through histology confirms the precision and repeatability of the protocol. Histological analysis of the joints, processed using the cutting–grinding technique and stained with Toluidine Blue, demonstrated the exclusive localization of particles within the synovial space in all five injected animals (5/5). This objective finding validates that the protocol reproducibly leads to successful intra-articular administration.

The combination of operative criteria (loss of resistance, absence of reflux) proved to be an effective method for this protocol. Although these are tactile criteria, their efficacy is objectively corroborated by the histological results, which showed no extravasation of the injected material. This validates their usefulness as a viable alternative to synovial fluid aspiration in this species.

The protocol constitutes a reliable methodological foundation for studies requiring the precise intra-articular administration of materials. The successful validation with particles generated in wear–corrosion processes positions it as a robust tool for the evaluation of materials at the micro- and sub-micro-scale, or for the induction of joint disease models in preclinical research, where precise localization is critical.

This work lays the groundwork for future standardization, although its immediate applicability is confined to the validated conditions. The validation was performed in adult male Wistar rats by a single operator, demonstrating high intra-operator repeatability. Generalization of the protocol to other conditions will require future inter-operator validation studies.

## Figures and Tables

**Table 1:** Example of recommended materials and instruments.

Category	Item	Specification	Reference/Manufacturer
Anesthesia	Isoflurane ≥ 99.9%	Induction 5%, maintenance 2%	Baxter Healthcare, Deerfield, IL, USA
Needle	27 G × ½” (0.4 × 12 mm)	Sterile, single-use	Nipro Medical Corporation, Osaka, Japan
Syringe	710LT SYR (100 µL)	High-precision for nano-volumes	Hamilton Company, Reno, NV, USA
Solvent	0.9% sterile NaCl	Vehicle for nanomaterial suspensions	Sigma-Aldrich (Merck KGaA), Darmstadt, Germany
Sterile Filter	0.22 µm (PVDF or PTFE)	Bactericidal microfiltration	MilliporeSigma (Merck KGaA), Burlington, MA, USA
Heating	Regulated heating pad	Maintenance at 37 °C	Harvard Apparatus, Holliston, MA, USA
Antiseptic	70–96% Ethanol	Preoperative skin cleansing	PanReac AppliChem (ITW Reagents), Darmstadt, Germany

**Note:** Commercial brand names are included for technical reference only and do not imply endorsement by the manufacturer. Suppliers should be adjusted according to local regulations and availability. LT (Low Taper): low-taper design; SYR (Syringe); PVDF (Polyvinylidene Fluoride); PTFE (Polytetrafluoroethylene). All described procedures are presented for methodological and reference purposes. Practical implementation must strictly comply with current national legislation, approval from the corresponding ethics committee, and international standards on animal welfare.

**Table 2:** Systematic Quantitative assessment of injection success and particle distribution.

Animal	Successful Injection	Extravasation	HPFs with Particles
R1	Yes	No	9
R2	Yes	No	8
R3	Yes	No	7
R4	Yes	No	9
R5	Yes	No	9
**Total**	**5/5**	**0/5**	**8.4 ± 1.1**

**Note:** Commercial brand names are included for technical reference only and do not imply endorsement by the manufacturer. Suppliers should be adjusted according to local regulations and availability. HPF (High-power field at 40× magnification).

**Table 3:** Systematic comparison of technical parameters in intra-articular injection protocols for the rodent knee.

Reference/Model	Injected Agent	Anatomical Landmark	Needle Specification	Injection Volume	Verification Method	Reported Success/Key Finding
Adaes et al., 2014 [1]	Collagenase (OA model)	“Knee joint space” (not further specified)	27 G	50 µL	Synovial fluid aspiration	Not explicitly reported; variable pathology onset.
Sang et al., 2023 [5]	Silver Nanoparticles (mouse)	Patellofemoral joint	30 G	20 µL	Joint mobility assessment	Not reported; therapeutic focus.
Aytekin et al., 2020 [22]	Saline (volume optimization)	Through the patellar ligament	27 G	25, 50, 100 µL	Observation of leakage, histology	Volume-dependent leakage: >50 µL caused leakage in ~40% of cases.
Wang et al., 2022 [21]	DPBS/Gadolinium (volume study)	Cranial margin of the patellar ligament	27 G	20, 30, 40, 50 µL	MRI for leakage, histology	Peri-articular leakage with 30, 40, 50 µL; no leakage with 20 µL. Recommended: 20–30 µL.
This study	CoCr-GO-HA wear particles	Medial border of the patellar tendon	27 G	35 µL	Loss of resistance + histological confirmation (Toluidine Blue)	5/5 success (100%), no extravasation. Combines precise landmark, safe volume, and direct verification.

**Note:** This comparison underscores the significant heterogeneity in anatomical descriptions, injection volumes, and verification methods across commonly cited protocols. Our proposed protocol addresses these critical gaps by (1) specifying a precise and palpable anatomical landmark (medial border of the patellar tendon), (2) employing a justifiable volume (35 µL) that resides within the safe range supported by empirical leakage studies [21], and (3) incorporating direct, objective verification via histological confirmation of particle localization.

## Data Availability

All data generated or analyzed during this study are included in this published article and Supplementary Materials.

## Supplementary Materials

The following supporting information can be downloaded at: https://www.mdpi.com/article/10.3390/nano16010009/s1. Figure S1: Inhalation anesthesia system with isoflurane. (**A**) Anesthetic induction with 5% isoflurane. (**B**) Anesthetic maintenance with isoflurane showing a transient adjustment to 2.6% during stabilization towards the target 2%. (C and D) Anesthesia chamber and complementary monitoring equipment; Figure S2: Microprecision instrumentation for intra-articular injection. Hamilton 710LT SYR syringe (100 µL) coupled to a sterile 27 G needle; Figure S3: Nipro^®^ 27 G × ½″ (0.4 × 12 mm) needle for intra-articular administration; Figure S4A,B: Surgical field preparation. (**A**) Shaving of the anterior knee area. (**B**) Skin disinfection with 70–96% ethanol; Figure S5: Positioning of the animal in dorsal decubitus with the posterior limb in slight flexion, showing accessibility to the knee joint; Figure S6: Identification of anatomical reference points through palpation: anterior tibial tuberosity, patellar tendon, and interarticular line.

## Abbreviations

The following abbreviations are used in this manuscript:

3Rs	Replacement, Reduction, Refinement
ARRIVE	Animal Research: Reporting of In Vivo Experiments
CEIC-R	Regional Clinical Research Ethics Committee (Comité Ético de Investigación Clínica Regional)
CoCr	Cobalt–Chromium (alloy)
GMA	Glycol Methacrylate
GO-HA	Graphene Oxide–Hyaluronic Acid
H&E	Hematoxylin and Eosin
MIA	Monoiodoacetate
MRI	Magnetic resonance imaging
OA	Osteoarthritis
PVDF	Polyvinylidene Fluoride
PTFE	Polytetrafluoroethylene
SECAL	Spanish Society for Laboratory Animal Science (Sociedad Española para las Ciencias del Animal de Laboratorio)
SPF	Specific Pathogen Free
TB	*Toluidine Blue*
