# Supplementary material for: Intra-Articular Injection in Wistar Rats: Standardization and Experimental Validation of a Precise Protocol for Nanomaterial Administration
Source: Nanomaterials (Basel). 2025 Dec 20;16(1):9. doi: 10.3390/nano16010009 (PMC12787652; doi:10.3390/nano16010009)
Supplement: Supplementary file 1 [file nanomaterials-16-00009-s001.zip › nanomaterials-4028567-supplementary.pdf]

SUPPLEMENTARY MATERIALS

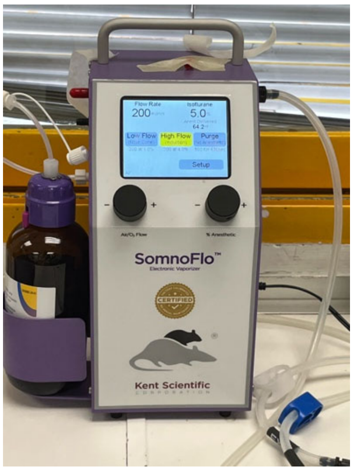

(a)

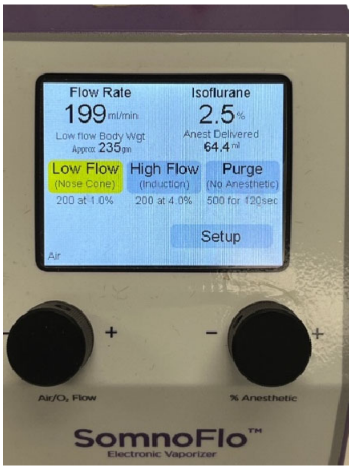

(b)

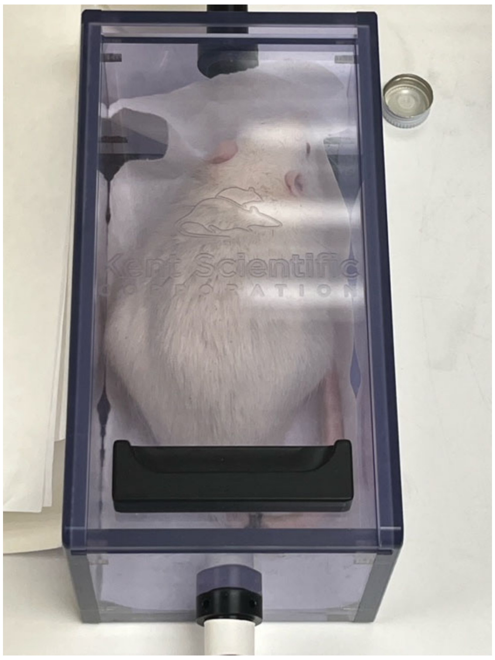

(c)

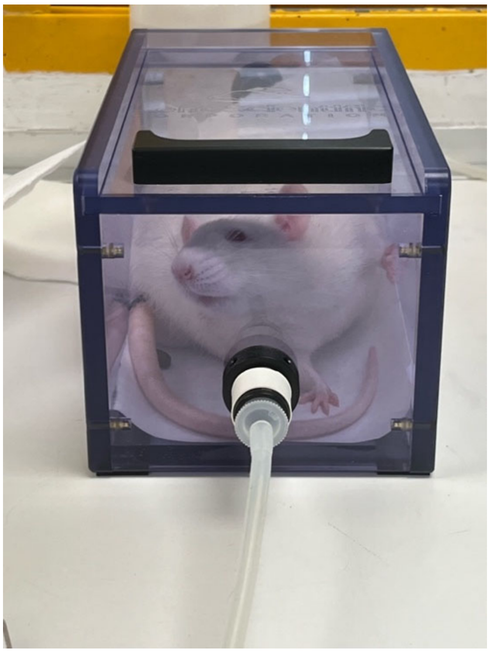

(d)

Figure S1. Inhalation anesthesia system with isoflurane. (A) Anesthetic induction with 5% isoflurane. (B) Anesthetic maintenance with isoflurane showing a transient adjustment to 2.6% during stabilization towards the target 2%. (C and D) Anesthesia chamber and complementary monitoring equipment.

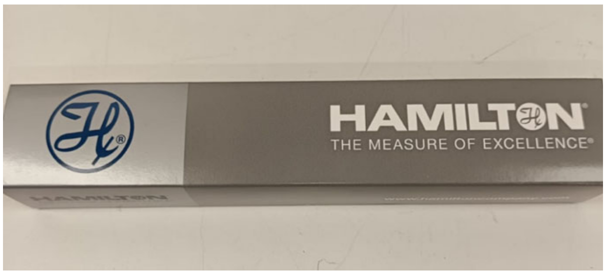

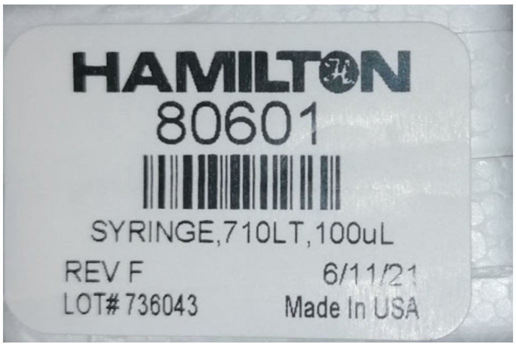

(**a**) (**b**)

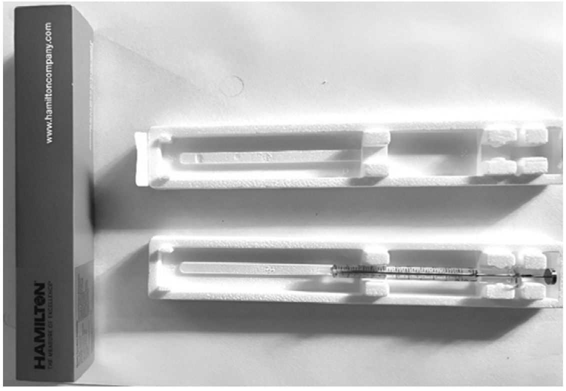

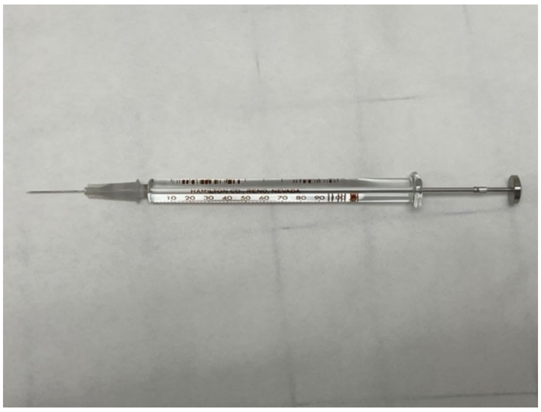

(c) **(d)**

Figure S2. Microprecision instrumentation for intra-articular injection. Hamilton 710LT SYR syringe (100 μL) coupled to a sterile 27 G needle.

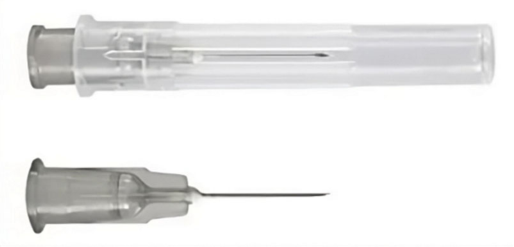

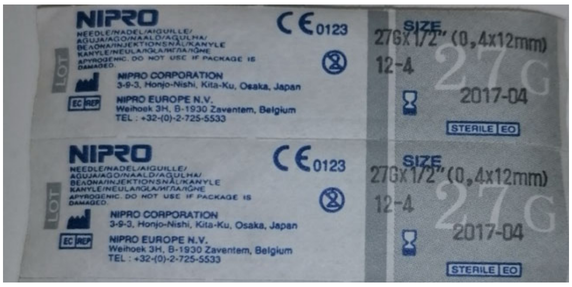

(a) (b)

Figure S3. Nipro® 27 G × ½″ (0.4 × 12 mm) needle for intra-articular administration.

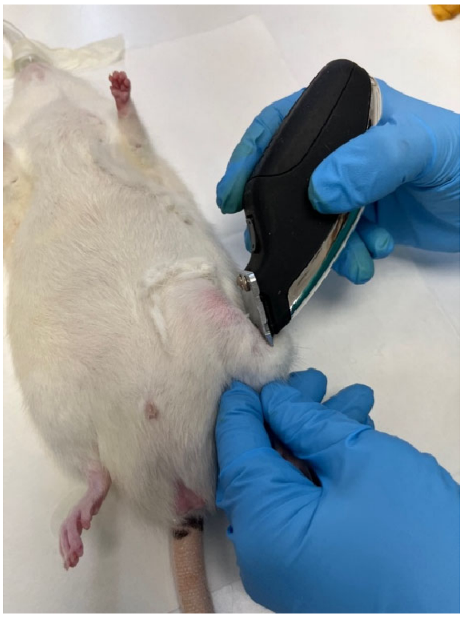

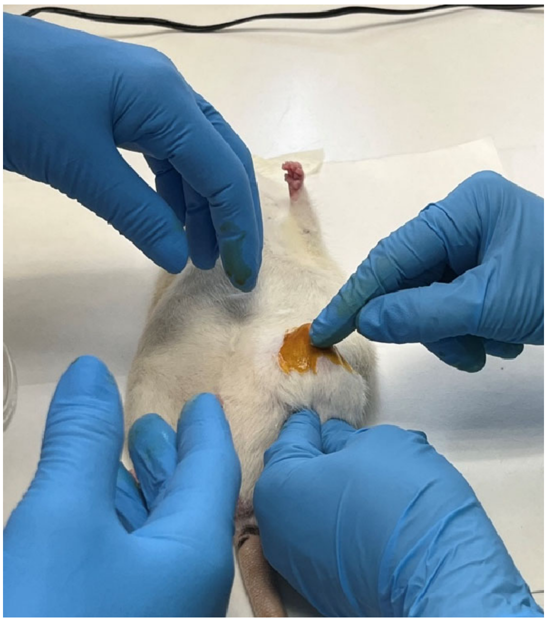

(**a**) (**b**)

Figure S4. Figure S4 A and S4B. Surgical field preparation. (A) Shaving of the anterior knee area. (B) Skin disinfection with 70-96% ethanol.

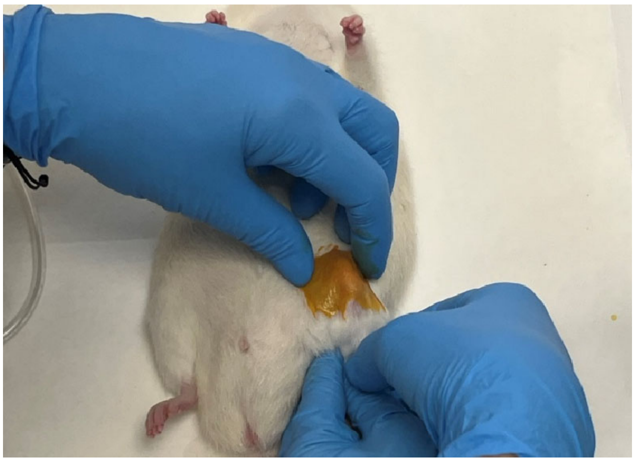

Figure S5. Positioning of the animal in dorsal decubitus with the posterior limb in slight flexion, showing accessibility to the knee joint.

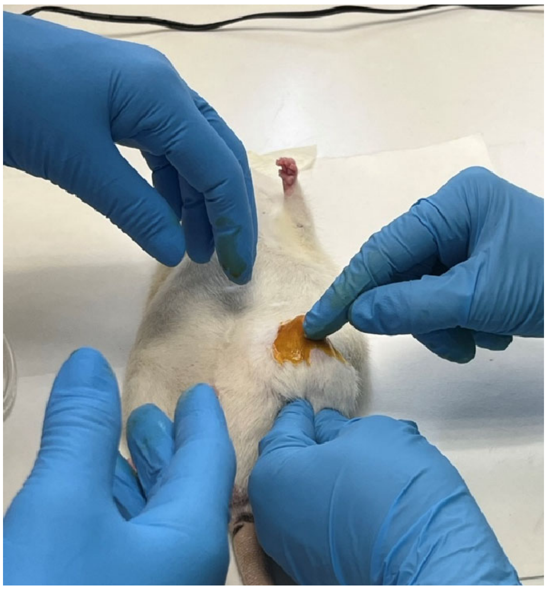

Figure S6. Identification of anatomical reference points through palpation: anterior tibial tuberosity, patellar tendon, and interarticular line.
